# Medicine

**Published:** 1896-06

**Authors:** George Parker


### Progress of the Medical Sciences.

### MEDICINE.

The discovery at Bath of **an organism** found almost invariably **in joints affected by rheumatoid arthritis**[1] is one of the highest importance, and calls for our hearty congratulations. The successful reproduction of the disease by inoculation, or its relief by an antitoxin prepared from the growth, will be eagerly looked for as the crowning proof in a laborious investigation.

Many observers have of late sought in vain for a similar cause for acute rheumatism, which presents many analogies to septic diseases; but though Leyden[2] has cultivated a microbe from **rheumatic endocarditis,** still that lesion is caused in so many different pathological circumstances that little importance can be attached to it. In speaking of this lesion we may refer to the practical results in preventing it recorded by Caton. The proportion of heart troubles can, he thinks, be greatly reduced by his method.[3] He not only insists on absolute rest with flannel bedclothes and salicylates, but as soon as the slightest blurring of the heart-sounds occurs, he applies a small blister over the apex of the heart, and, as the effects of that pass away, another close to it, and so on, until with absolute rest and the addition of iodides to the salicylates the murmur disappears, as it generally does if this treatment is persevered with long enough.

In gout, too, the question of an auto-intoxication from defective metabolism as opposed to a microbial poison causing lesions in which urates are passively deposited is hotly discussed. Thus salicylates have been given as a germicide and again as increasing the excretion of uric acid; but Bohland shows that, in health at least, the increased excretion of uric acid is due to their causing a great increase in the white corpuscles.[4] This, of course, lends

[1] See p. 123 of this number. [2] *Deutsche med. Wchnschr.*, 1894, xx. 913.
[3] *Liverpool M.-Chir. J.*, 1895, xv. 325; abstract in *Med. Rec.*, 1895, xlviii. 748.
[4] *Centralbl. f. innere Med.*, 1896, xvii. 70; see *Edinb. M. J.*, 1896, xli. 871-2.

force to the view that uric acid is chiefly formed from broken-down leucocytes. Klemperer,[1] Berkart, and others regard the lesions in the joints as produced by some unknown agent, and the uric acid deposited there passively. The former shows that the supposed excess of the acid in gout exists in other diseases, as in leukæmia, and is not the cause of the deposition, for gouty blood is far from saturated and can dissolve much more than is present; while Neusser finds an excess of leucocytes in the same blood.

Besides the production of disease by the schizomycetes, other parasites play an important part. Thus the **Tsetse fly-disease**, which destroys the cattle and horses over immense districts in Africa, has recently been shown to be probably due to a plasmodium which is merely conveyed by the fly. When introduced into a suitable animal it multiplies enormously, so that over 300,000 have been counted in a cubic millimetre, and it forthwith commences to destroy the red corpuscles, producing a profound and fatal anæmia. However, the plasmodium is rarely, if ever, pathogenic to man, any more than the germ of **Texan cattle fever** which seems to be similarly conveyed by certain lice.[2] It is otherwise with the plasmode of **malaria**, which destroys more human beings than any other disease except, perhaps, phthisis. In malaria and phthisis then we have the two great types of these **diseases**, the **plasmodial** and the **bacterial.** The latter has been long studied, and we are in theory at least able to combat it by vaccinations and antitoxic serums. In the former nothing of the kind has been found effective. In America the plasmode has been discovered universally in every well-marked case of malaria, and most European and Indian observers have found it in the great majority of their cases; but Lawrie[3] almost alone denies its specific origin and looks upon it as a retrograde product of the blood-cells. He denies that it is to be found in many of the worst cases of malaria. He points out that it has never been isolated in a pure culture, though Manson claims to have produced a malarial attack and vast multitudes of the plasmodes in the body by injecting a minute quantity of infected blood.

Lawrie answers this by saying that the injection of healthy blood is normally followed by a febrile attack. Moreover, he insists that the "swarming" movement of Laveran's corpuscles can be easily seen in the white corpuscles of man, and the flagellate and ciliary bodies are, he thinks, due to ordinary changes in these protoplasmic bodies. A much more doubtful part of his criticism is based on his observations on the history of white corpuscles in frogs' blood. These he finds are at first intra-corpuscular, and later on free and able to change them-

[1] *Deutsche med. Wchnschr.*, 1895, xxi. 655; abstract in *Med. Chron.*, 1896, N.S. iv. 116
[2] *Nature*, 1896, liii. 566. [3] *Brit. M. J.*, 1896, i. 1135.

selves into crescents and other orms. Thus every characteristic of Laveran's cells has its analogue in the blood-cells of man or reptiles. On this theory malaria is an endemic disease of the spleen, akin to that of the thyroid, and the so-called organisms are the degenerate, atavistic blood-cells which the diseased organ manufactures and which have reverted to reptilian types. The periodic fever is an exaggeration of the effects of the diurnal activity of the spleen, which activity, with its nocturnal inactivity, is in health the cause of the variation of the morning and evening temperatures. In disease this may give a quotidian, tertian, or other paroxysm. Finally, when the splenitis is chronic and profound, the functions of the spleen are in abeyance; even Laveran's corpuscles are not produced, and permanent failure of nutrition and anæmia follow.

Though we must hesitate before accepting chronic splenitis as a cause of the anæmia in malaria, or indeed Lawrie's strange views as to the origin of reptilian white corpuscles, his argument is singularly ingenious, and the criticism is perfectly just that the canons of proof on behalf of Laveran's corpuscles are far from complete. It is probably true on either hypothesis that the anæmia, at any rate in the early stages of the disease, is due to the destruction of red corpuscles, and not to their insufficient production.

Manson[1] confesses that objections will have weight against the plasmodium until the disease is communicated by the administration of plasmodial forms which have, for instance, been passed through the body of the mosquito, for this he regards as the carrier of the organism and its intermediate host. He has not indeed traced it into the tissues of that insect, but he and Surgeon-Major Ross have shown that the crescents when swallowed by the mosquito, instead of being killed and digested, rapidly develop in its stomach and become first spheres and then flagellated as if in preparation for extra-corporeal life. He draws with wonderful skill the various analogies between the known course of the filaria sanguinis hominis in its passage through the mosquito and what he believes to be the development of the malarial plasmode; and clearly the decision of the question whether Laveran's cells are abnormal body-cells, or a foreign invader, must depend on the possibility of tracing it through the mosquito or a similar host. Lawrie's theory indeed would add another gland to those whose mysterious functions have been so much invoked of late.

Whatever be the true pathology, one thing seems pretty clear, that practically the detection of Laveran's cells is of the highest importance. Our American brethren and Ross[2] have already shown that obscure cases supposed to be bronchitis or typhoid could be diagnosed and cured when these bodies were discovered in the blood. Besides there is reason to think

[1] *Lancet*, 1896, i. 833. [2] *Brit. M. J.*, 1896, i. 260, 293.

that cases of malaria are still to be found in rural districts in England, so that the question is not only of academic interest.

The functions of the spleen with regard to another disease, **lymphadenoma**, have been decided in a manner analogous rather to the views of Manson than to those of Lawrie. The actual bacillus has been found in the spleen of a patient, cultivated and injected into an animal in which the disease was reproduced, and from the new growths in the glands of this dog a fresh culture of the same bacillus was obtained by Dr. Delbet.[1]

In a similar way the cause of **leucocythæmia** has, it is said, been traced. The enlargement of the spleen in this disorder appears to be a secondary phenomenon, for it is often absent in those acute forms which are fatal in a few days, and of which many instances have of late been given. In the discussion at the London Meeting, W. G. Spencer asserted the unity of several of these blood diseases, holding that just as we get general tuberculosis, strumous glands and phthisis from a common cause; so we find a generalised lymphadenoma, or lymphomas producing leucocythæmia and other forms. Here Delbet's bacillus would take the place of Koch's as the connecting irritant. However, we must not be carried away by the many clinical resemblances to septic diseases. For in the latter the leucocytosis is due to the increase of polynuclear cells, whereas in leucocythæmia the bulk of the cells consists of mononuclear ones. This is especially the case in the acute type as Fraenkel notices,[2] where the polynuclear ones may be absolutely diminished.

In **typhoid fever** we have often a difficulty in making our diagnosis at an early stage, and often too an almost equally serious one in deciding when patients cease to be infectious; thus instances are known where a convalescent patient has spread the complaint among others. Elsner's[3] test for the presence of the typhoid bacillus by plate cultivations on a gelatinised emulsion of potato to which one per cent. of potassium iodide had been added, enables us to discover with ease whether it exists in the fæces or not. The typhoid bacillus and the bacterium coli are the only ones which survive, and the former produces circular transparent droplets on the plate in forty-eight hours. The difficulty of finding a trustworthy mode of distinguishing the typhoid germs has hitherto been a great practical difficulty.

We need, however, to remember that there are other means of infection besides fæcal contamination. The urine appears to be a possible channel, for Wright and Semple found active bacilli in the urine of six out of seven cases,[4] and they remark on

[1] *Med. Week*, 1895, iii. 307.
[2] *Deutsche med. Wchnschr.*, 1895, xxi.; abstract in *Med. Chron.*, 1896, N.S. iv. 267.
[3] *Lancet*, 1896, i. 73. [4] *Lancet*, 1895, ii. 196.

the proof this fact affords of the septicæmic nature of the disease and the distribution of the infection through the whole of the blood.

The end of the infective stage again, in some cases, must be put long after the urine and fæces are free of germs, for they can be found in suppurative discharges for an unlimited period, notably for years when a sinus has formed after osteomyelitis.

It has been suggested that the delicate modern tests for albumose would show the continuance of infectiveness, since so long as bacterial metabolism or suppuration goes on, so long will surplus albumoses be excreted by the kidneys. Harris,[1] of Chicago, finds such a test in a saturated solution of salicyl-sulpho-tungstate of sodium, a few drops of which form a cloudiness in urine if any albumoses are present. Of course all albumen must be first precipitated and removed. Harris insists that the so-called peptonuria, or albumosuria, is simply a symptom of bacterial peptonising action and may be often taken as a test of the severity or mildness of the attack. It does not, however, seem at all clear why this reaction is so fortunately absent from the strivings of the bacillus coli and yet appears in exact proportion to the severity of typhoid or of suppuration.

It may be interesting to notice here that the typho-toxin, according to Bokenham and Soltau Fenwick,[2] consists of an albumose which they found in the spleen of patients dying in the height of the disease, and which when injected into animals produced pyrexia, loss of flesh, and anorexia.

The **diazo-reaction** is growing in favour as a clinical test, though it has many limitations and is found in certain other diseases, notably pneumonia. Among other precautions, Hewlett remarks that the urine and the sodium nitrite must be fresh, and that the latter must not be in excess. The mixture must be made alkaline, and the resulting colour-reaction should be a deep red and the foam a definite salmon colour, if typhoid is really present.[3] Of course no reaction is found either at a very early or at a late stage of the disease.

One other symptom of typhoid of some value in prognosis is to be found in the **ulcers** not infrequently seen **on the palate and tongue**. These are said to run a similar course to those of the intestines and show by their healing or the opposite the progress made in the intestinal ones.[4]

Nothing very new has been proposed in the way of **treatment.** Various attempts have been made to show that a less restricted diet is desirable, and that meat extracts, milk sugar, somatose, and similar substances may be added to the milk diet with advantage, while on the other hand Lesser[5] and others report satisfactory results when the patients took nothing whatever but water, in the worst period of the disease, for days together.

[1] *Am. J. M. Sc.*, 1896, cxi. 557. [2] *Brit M. J.*, 1895, i. 801.
[3] *Brit. M. J.*, 1896, i. 136. [4] *Brit. M. J.*, 1896, i. Epitome, p. 45.
[5] *Med. Rec.*, 1895, xlviii. 541.

The cold bath treatment has been opposed by the advocates of the external use of guaiacol (10 to 20 drops painted on the skin and covered over), but symptoms of collapse after it are not unknown. Its recent successful use in rheumatoid arthritis suggests that it possibly may have an inhibitory action on the bacillus. Many of the supporters of Brand's treatment now claim that the mortality is reduced to three per cent., and Brand himself says that in 1,200 of his cases none died who were bathed before the fifth day. A discussion of the subject at Birmingham showed some difference of opinion. The records at the General Hospital, for ten years, give a mortality of 16.8 per cent.; but this appears largely owing to the late date at which patients come in. Thus Saundby was able to show a death-rate of only 6.5 in his more recent ones, and bathing is confessedly of little use unless begun early. Simon thinks that the constant care and observation necessitated by bathing have much to do with the success claimed for it.[1] It is probable too that the rapid excretion as shown by the toxicity of the urine under bathing has more to do with the result than the lowered temperature. Jacquet also proves that, in typhoid, the red corpuscles tend to leave the general circulation and to accumulate in the spleen and glands; but with cold baths this anæmia is overcome and the increase of corpuscles in the general circulation is enormous.[2] Similar results are aimed at by some American physicians, who, besides preliminary doses of calomel, administer as much cold water as the patient will drink, and wash out the bowels at frequent intervals with cold or tepid water.

The **Vaccination controversy** has been unusually active. The most striking part of it is the assumption by its opponents that abiogenesis of the small-pox virus exists or at any rate that the virus has a normal *habitat* outside the body in decaying matter, for which there does not appear a particle of evidence. Truly the phrase "insanitary conditions" is mighty to conjure with. The controversy will never be set at rest until the small-pox virus is isolated in a pure cultivation and the action of vaccine explained. Hence the work of Monckton Copeman bids fair to be most important. He and Klein discovered minute bacilli in early stages of vaccinia and of variola. After numerous attempts he succeeded in inoculating those obtained from small-pox into hens' eggs, the outside of which were carefully sterilised, and these he kept for a month at a temperature of 37°. He found in them a pure cultivation of the bacillus, with which he vaccinated calves and obtained vesicles, from which in turn he vaccinated himself and several children with complete success.

[1] *Birmingh. M. Rev.*, 1896, xxxix. 262.
[2] *Med. Chron.*, 1896, N.S. iv. 148.

There are certain possible fallacies in the method employed which render it most important that these experiments should be verified without delay, and on this he is now engaged.[1]

An interesting discussion took place at Edinburgh on a paper by J. C. Dunlop, respecting the significance of **oxaluria.** He showed that oxaluria could be easily produced in health by adding oxalates to the food and that it stopped when the diet contained none.[2] Moreover, he denied that oxaluria was a sign of perverted metabolism or disease of any kind, though the passage of oxalates may be painful. Finally he disposed of Begbie's oxaluria as a form of acid dyspepsia. We may, however, say that though he showed that oxaluria was often a normal process, further observation is needed to show that it never occurs as a result of diseased metabolism.

George Parker.

### SURGERY.

[1] *Practitioner*, 1896, lvi. 475-9.

[2] *J. Path. & Bacteriol.*, 1896, iii. 389; abstract in *Edinb. M. J.*, 1896, xli. 634.

